# Establishing a scalable perfusion strategy for the manufacture of CAR‐T cells in stirred‐tank bioreactors using a quality‐by‐design approach

**DOI:** 10.1002/btm2.10753

**Published:** 2025-01-28

**Authors:** Tiffany Hood, Pierre Springuel, Fern Slingsby, Viktor Sandner, Winfried Geis, Timo Schmidberger, Nicola Bevan, Quentin Vicard, Julia Hengst, Noushin Dianat, Qasim A. Rafiq

**Affiliations:** ^1^ Department of Biochemical Engineering University College London London UK; ^2^ Product Excellence Bioreactor Technology Sartorius Stedim UK Limited Epsom UK; ^3^ Digital Solutions Sartorius Stedim Austria GmbH Vienna Austria; ^4^ Digital Solutions Sartorius Stedim Biotech GmbH Goettingen Germany; ^5^ BioAnalytics Application Development Essen BioScience Ltd. (part of the Sartorius Group) Royston UK; ^6^ Cell Culture Technology Marketing Sartorius Stedim France S.A.S. Aubagne France; ^7^ Cell Culture Technology Marketing, Sartorius Stedim Biotech GmbH, Goettingen, Germany

**Keywords:** CAR‐T, perfusion, immunotherapy, process control, process intensification, quality‐by‐design, stirred‐tank bioreactor

## Abstract

Chimeric antigen receptor T cell (CAR‐T) therapies show high remission rates for relapsed and refractory leukemia and lymphoma. However, manufacturing challenges hinder their commercial viability and patient accessibility. This study applied quality‐by‐design principles to identify perfusion critical process parameters for CAR‐T expansion in stirred tank bioreactors to maximize yields. A design of experiments in the Ambr® 250 High Throughput Perfusion small‐scale bioreactor revealed that earlier perfusion starts (48 h vs. 96 h post‐inoculation) and higher perfusion rates (1.0 VVD vs. 0.25 VVD) significantly increased cytotoxic CAR‐T cell yields without compromising critical quality attributes. Optimizing perfusion improved growth kinetics and yields across donor samples, achieving densities >21 × 10^6^ cells/mL in 7 days, outperforming traditional fed‐batch and static flask cultures. This study underscores the importance of optimizing perfusion parameters to maximize CAR‐T yields and quality and highlights the utility of scale‐down models in reducing time, costs and risks associated with process development.

**Translational Impact Statement**

The expansion of CAR‐T cells to reach numbers sufficient for a clinical dose represents the longest phase in existing CAR‐T manufacturing processes. By optimizing perfusion parameters, this work demonstrates that CAR‐T growth kinetics and yields can be significantly increased, without adversely impacting final product critical quality attributes. Implementation of such optimized perfusion feeds could therefore contribute to reducing overall CAR‐T manufacturing wait times and costs.

## 1 INTRODUCTION

While initial CAR‐T treatments have shown significant clinical success, production and commercialization of these products can provide equally significant challenges.^1^ Large‐scale manufacturing of these therapies must be optimized to succeed commercially. The current high cost, rate of manufacturing failures, and manufacturing turnaround time will not be sustainable when these therapies scale to larger patient populations and additional disease indications.^2^

The ex vivo cell expansion of CAR‐T cells is a requirement for all of the FDA‐approved CAR‐T cell products and the majority of academic clinical processes currently in development.^3^ The expansion step is required to generate a sufficient number of cells to meet the dose specifications. However, manufacturing protocols and expansion platforms for even the FDA‐approved products often differ, and there is reliance upon manual technologies, such as tissue culture flasks and culture bags and/or platforms that offer limited process monitoring and control capability.^4^ Whilst such systems platforms are often lower cost and present a low barrier to entry, they are limited in terms of scalability and industrialization. In contrast, stirred‐tank bioreactors (STRs) are a cell expansion technology widely used across the pharmaceutical and biopharmaceutical sectors for the production of both small molecule and cell‐derived biotechnology products.^5^, ^6^ These systems not only have proven scalability but are equipped to enable extensive process monitoring and control capability, with the ability to control key culture parameters such as pH, dissolved oxygen, and temperature amongst other parameters. Moreover, such systems are capable of operating under different modes of operation including fed‐batch and perfusion. These controlled bioreactor systems provide more homogeneous environments than static flasks by controlling culture parameters such as agitation, pH, and dissolved oxygen (DO) levels. Incorporation of perfusion capabilities enables increased control of feeding strategies and ensures continuous nutrient availability. This process intensification can enable high cell densities to be reached within a much smaller bioreactor footprint compared with other fed‐batch approaches.

Perfusion feeding regimes have been previously utilized for the expansion of T cells in the WAVE™ bioreactor platform.^7^, ^8^ In another study, Smith et al. implemented perfusion in the Xuri rocking motion bioreactor resulting in T cell yields that were more than twice as high compared to growth without.^9^ Similarly, Gatla et al., (2022) also showed the feasibility of implementing perfusion in a STR for improving T cell yields.^10^ However, these studies did not investigate whether and how separate perfusion process parameters impacted culture performance differently. Additionally, as existing perfusion studies have primarily focused on T cell growth, the effects of high cell density perfusion cultures on CAR‐T functionality and CQAs remain to be determined.

In this study, we examined the intensification of CAR‐T expansion in a perfusion STR by investigating the impact of key perfusion parameters using a quality‐by‐design (QbD) approach. The first part of this work aimed to characterize two perfusion critical process parameters (CPPs) using a design of experiments (DOEs) study in Ambr® 250 High Throughput Perfusion single‐use bioreactors. The impacts of key perfusion parameters on CAR‐T cell yield, phenotype, and cytotoxicity were investigated as relevant CQAs for the production of clinically relevant CAR‐T cells.^11^, ^12^ Additionally, the impact of donor variability on the effect of these perfusion regimes was investigated. The optimized perfusion parameters were then identified and compared to a fed‐batch bioreactor process and flask culture to determine if optimizing perfusion can improve CAR‐T expansion process efficiencies.

## 2 RESULTS

### 2.1 DOEs study to investigate perfusion process parameters for stirred‐tank bioreactor (STR) studies

The first phase of work aimed to determine the effects and interactions of potential CPPs related to perfusion on final CAR‐T cell yield and quality. A DOE study was established to investigate critical parameters and how they interact so that the optimal levels of each parameter could be selected. Three experimental factors and levels were selected to be tested in the DOE: (i) perfusion start time (48, 72, and 96 h post‐inoculation), (ii) perfusion rate (0.25, 0.5, 1.0 VVD), and (iii) donor (1–3) (Table 1). These ranges of perfusion parameters were chosen as representative intervals to capture the effects of perfusion initiated at different time intervals and at different rates. The goal was to determine which parameters within the experimental space maximized cell yield while retaining cell function. Statistical models were created using the experimental data for each cell yield or quality response to understand the effect of each factor tested within the DOE design space.

**TABLE 1:** Factors and levels tested in the perfusion DOE included feed start day, perfusion rate, and donor.

Parameter	Levels		
Perfusion start time	48 h	72 h	96 h
Perfusion rate (VVD)	0.25	0.5	1
Donor	1	2	3

**FIGURE 1:**
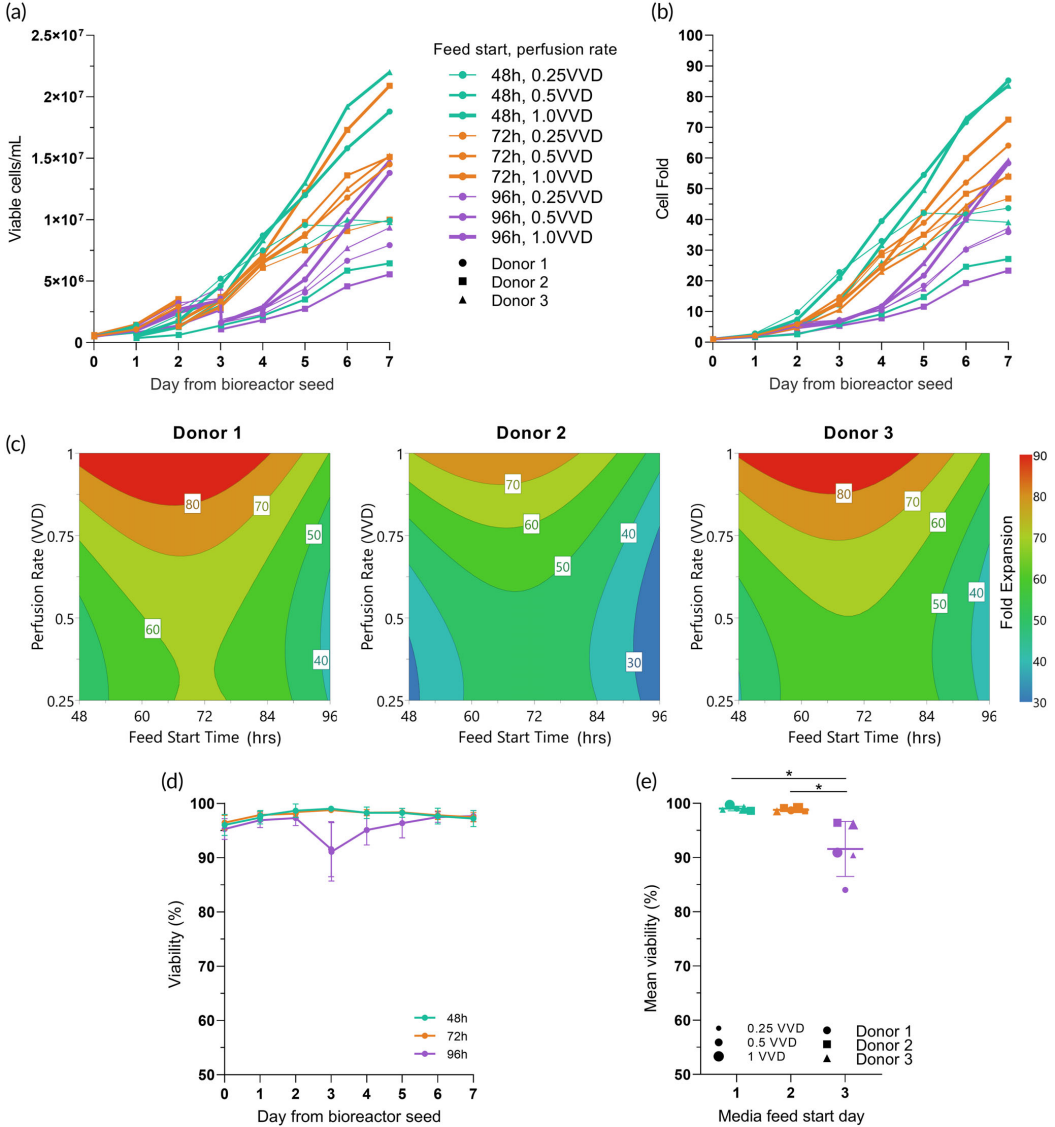
Impact of perfusion start time and perfusion rate on cell growth and viability in the Ambr® 250 DOE. (a) Viable cell density by day. (b) Cell fold by day. (c) DOE contour plots representing the effects of perfusion parameters on cell fold per donor. (d) Impact of perfusion start time on average cell viability by day and (e) on day 3 post‐inoculation. Data represents *n* = 15 perfusion DOE conditions. Error bars represent standard deviation; *p < 0.05. VVD, vessel volumes per day.

The impact of the DOE factors on CAR‐T cell growth and viability is illustrated in Figure 1. As expected, there were significant differences between the different experimental conditions, resulting in a range of viable cell densities from ~5.6 to 22.5 × 10^6^ cells/mL (Figure 1a), corresponding to a cell fold increase ranging from 23.3 to 85.3, respectively (Figure 1b). The greatest CAR‐T yield was achieved with donor 3 when perfusion was started at the earliest tested start time (48 h post‐inoculation) and at the highest tested perfusion rate (1.0 VVD). The experimental condition that resulted in the lowest yield and cell fold increase was the perfusion start time of 96 h, perfusion rate of 0.5 VVD, and with donor 2. This broad range observed in the cell growth data is unsurprising and demonstrates that the perfusion process parameters investigated have a significant impact on CAR‐T growth yield.

To further understand the effects of the DOE factors on cell fold expansion at the end of bioreactor culture, a linear regression model for fold expansion was created. Perfusion start time, perfusion rate, and donor variability were found to all significantly impact CAR‐T fold expansions (Supplemental Figure 1a, b). Perfusion rate, however, was found to have the most important influence on fold expansion, almost 3‐fold higher than perfusion start time and donor variability (Supplemental Figure 1c). Generated contour plots highlight that higher fold expansions (>75) could be achieved despite inter‐donor differences if a minimum perfusion rate of 0.85 VVD starting from 48 to 72 h post‐inoculation was applied (Figure 1c). However, the small but significant differences in the optimal ranges for maximizing fold expansions, as shown for each donor in the contour plots (Figure 1c), and the inclusion of donor as a significant parameter in the regression model (Supplemental Figure 1b, c), suggest that fold expansions could potentially be further enhanced by optimizing perfusion feeds on a per‐donor basis. The generated model for fold expansion was significant (*p* < 0.0001) and plotting the predicted versus observed fold expansion values demonstrated a strong model fit (*R* ^2^ = 0.97, *Q* ^2^ = 0.88) (Supplemental Figure S1).

Cell viability during the culture is also a critical response to monitor. All bioreactor cultures in the DOE started and finished with a viability above 95%. However, the experimental conditions that had a perfusion start time of 96 h after inoculation start resulted a drop in viability on day 3 of the culture (Figure 1d), dropping to 91.6% ± 5.1%. This is in contrast to the 48 and 72 h perfusion start times where the viability was consistently above 95%. However, after perfusion was started, it is notable that the viability recovered to 94.5% by day 4 of culture, 96% viability by day 5, and then 97% by 6 where it remained until the end of the culture at day 7. In all cases, the cell viability remained above 80% with the viability of the conditions with a perfusion start time of 96 h averaging 91.6% ± 5.1% viable cells on day 3 of culture (Figure 1e).

### 2.2 Culture monitoring correlates with cell growth

**FIGURE 2:**
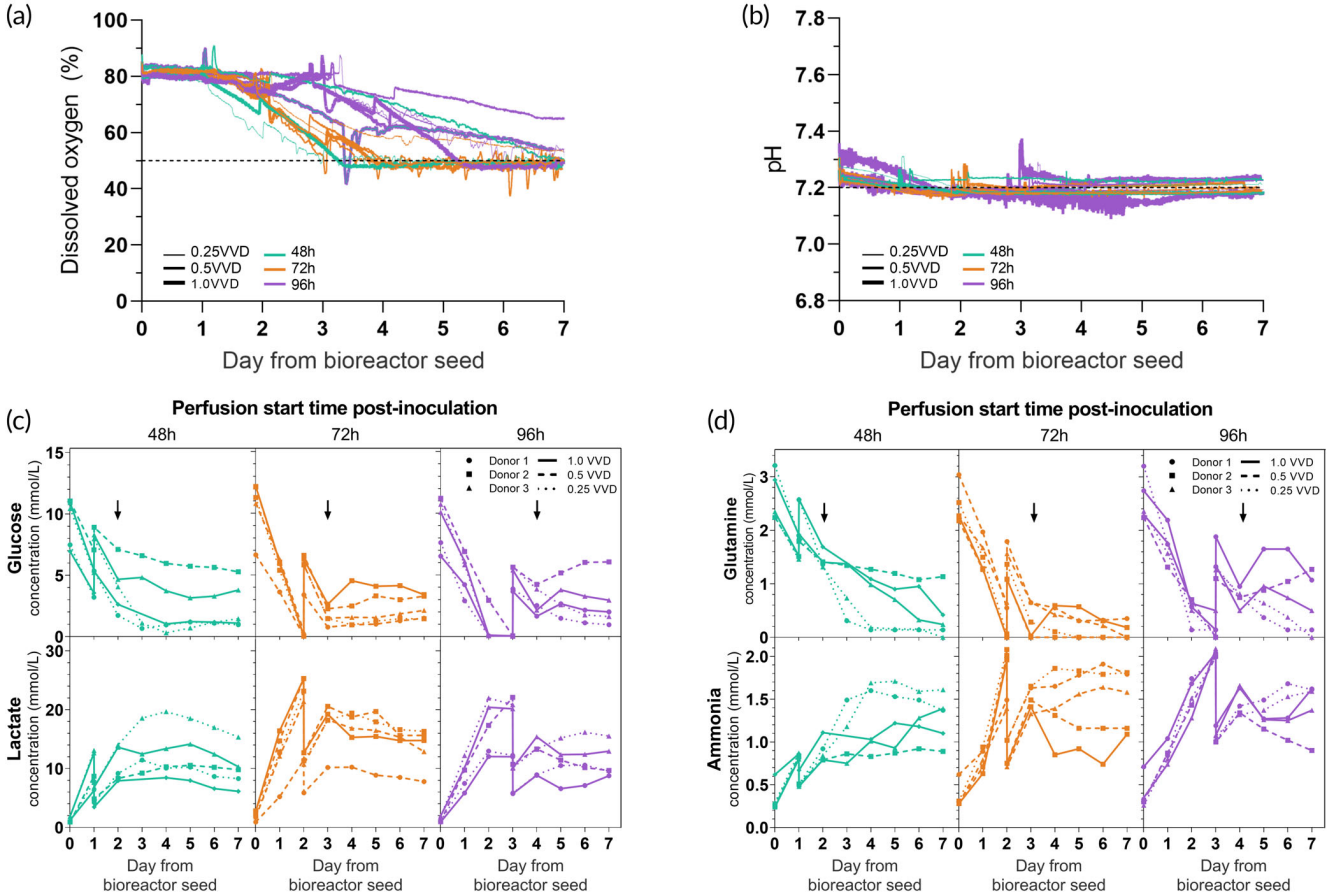
Culture trends including pH, DO, and metabolite concentrations impacted by DOE perfusion parameters. (a) Dissolved oxygen (DO) and (b) pH over time in the Ambr® 250 perfusion cultures. Black dashed lines represent parameter setpoints. (c) Impact of perfusion parameters on daily glucose and lactate, and (d) daily glutamine and ammonia concentrations (mmol/L). Arrows represent onset of perfusion. Data represents *n* = 15 perfusion DOE conditions. VVD, vessel volumes per day.

The pH, DO, and metabolite levels were monitored throughout the culture. The pH and DO levels were allowed to drift down naturally before being generally maintained within the setpoints of 7.2 ± 0.05 and 50%, respectively, despite large differences in perfusion regimes and subsequent cell densities within the DOE. Spikes in pH and DO were observed on days of media additions and onset of perfusion (Figure 2a, b). The conditions that reach DO setpoint first generally correlate with the conditions with the fastest initial growth, thereby increasing oxygen usage.

Depletion of energy sources, glucose and glutamine, and accumulation of waste products, lactate, and ammonia, were also correlated with the perfusion parameters tested in the DOE, in particular the perfusion start time. Spikes in metabolites were also observed and corresponded with the perfusion start which resulted in replenishment of glucose and glutamine and dilution of lactate and ammonia (Figure 2c, d). The depletion of glucose in the medium was primarily dependent on the perfusion start time. The conditions with a perfusion start time after 96 h had sustained depletion of glucose between days 2 and 3 of culture, while the other conditions did not (Figure 2c). Similarly, accumulation of lactate was also predominantly dependent on perfusion start time. Cultures with a perfusion start time at 48 or 96 h after inoculation showed higher accumulation of lactate beyond 20 mM until the respective onset of perfusion.

Glutamine depletion was impacted by both the perfusion start time and the perfusion rate. Glutamine was depleted between days 2 and 3 for cultures that had a perfusion start time 96 h after inoculation but were not for the earlier perfusion start times. Glutamine concentrations were also found to be below limit of detection between days 4 and 7 for cultures that had a perfusion rate of 0.25 VVD or 0.5 VVD, while cultures with a perfusion rate of 1.0 VVD were not depleted of glutamine until days 6–7 (Figure 2d). Ammonia showed some variability in accumulation that was correlated to the perfusion strategy (Figure 2d). This is likely because ammonia is produced as a by‐product during the breakdown of glutamine.^13^ However, it never reached levels above 2.4 mM.

### 2.3 Cell quality characteristics associated with donor

**FIGURE 3:**
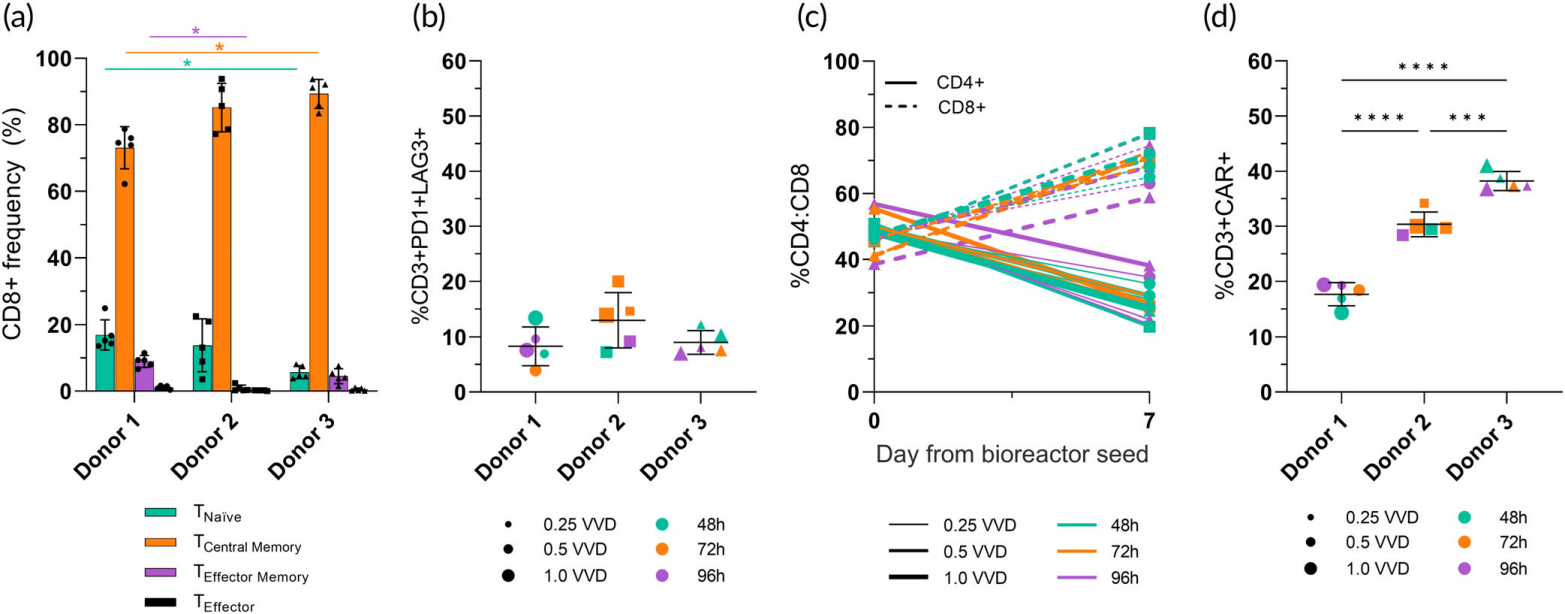
Cell quality markers impacted by donor in the perfusion DOE. (a) CD8+ T cell differentiation marker expression at the end of the 7‐day bioreactor perfusion cultures was broken into four populations: %CD8+CD45RO‐CCR7+ (naïve); %CD8+CD45RO+CCR7+ (central memory (CM)); %CD8+CD45RO+CCR7‐ (effector memory (EM)); and %CD8+CD45RO‐CCR7‐ (effector). (b) PD1+ and LAG3+ exhaustion marker expression at the end of the 7‐day bioreactor on CD3+ T cells. (c) CAR expression percentage in harvested CD3+ T cells. (d) Changes in CD4:CD8 ratio from day 0 – 7. Data represents n=15 perfusion DOE conditions. Error bars represent standard deviation; *p < 0.05, ***p < 0.001. ****p < 0.0001 VVD=vessel volumes per day.

The differentiation and exhaustion marker expression of the cells was also monitored during the expansion as these are indicative of CAR‐T cell function.^11^ Although significant inter‐donor differences were found in differentiation marker expression at the time of harvest, the investigated perfusion parameters were not found to significantly impact the expression of these markers. By harvest, approximately ~18% of harvested Donor 1 T cells expressed naïve differentiation markers on average, significantly more than Donor 3 with 5.7% ± 1.9%, respectively (*p* = 0.03) (Figure 3a). Donor 1 consequently had approximately 75% of cells expressing central memory markers CD8 + CD45RO + CCR7+, in lower proportion compared to Donor 2 and Donor 3, 85% and 90%, respectively. Over the 7‐day expansion period, the expression of naïve differentiation markers was generally found to increase over time in all donors (Supplemental Figure 2a). Regardless of donor or tested perfusion parameters, over 90% of harvested cells expressed either naïve or central memory markers following the 7‐day perfusion expansion in the Ambr® 250 bioreactor.

With regards to cellular exhaustion, the simultaneous expression of exhaustion markers PD‐1, and LAG‐3 by CD3+ T cells was found to range from 3.9% to20% by day 7 post‐bioreactor seed for all 15 tested perfusion conditions (Figure 3b). Neither perfusion parameters nor donor was found to significantly impact the level of exhaustion by harvest, even in cultures sustaining the highest final cell densities. Characterization of exhaustion marker expression for CD4+ and CD8+ T cell subsets separately, revealed slightly higher co‐expression of PD1+ and LAG3+ in the CD4+ subpopulation though this difference was only found significant for donor 2 (Supplemental Figure 2b). In all experiments, the majority of harvested cells were CD8+, with CD4:CD8 found to shift from 1:1 to approximately 1:2.5 following the 7‐day expansion (Figure 3c). Finally, perfusion parameters were not found to significantly alter CAR expression at harvest, which ranged from 20% to 40%, due to significant inter‐donor differences (Figure 3d).

### 2.4 Perfusion bioreactor process generates significantly higher cell densities compared to fed‐batch bioreactor and static T‐flask culture processes

The findings from the DOE study showed that, in general, a perfusion start time of 48 h post‐ inoculation and a perfusion rate of 1.0 VVD supported the highest cell growth without adversely impacting cell quality. The next phase of work, therefore, compared the best experimental perfusion condition (perfusion start time 48 h after inoculation and a perfusion rate of 1.0 VVD) with a fed‐batch bioreactor process and a static T‐flask culture with all three donors over a seven‐day culture period.

**FIGURE 4:**
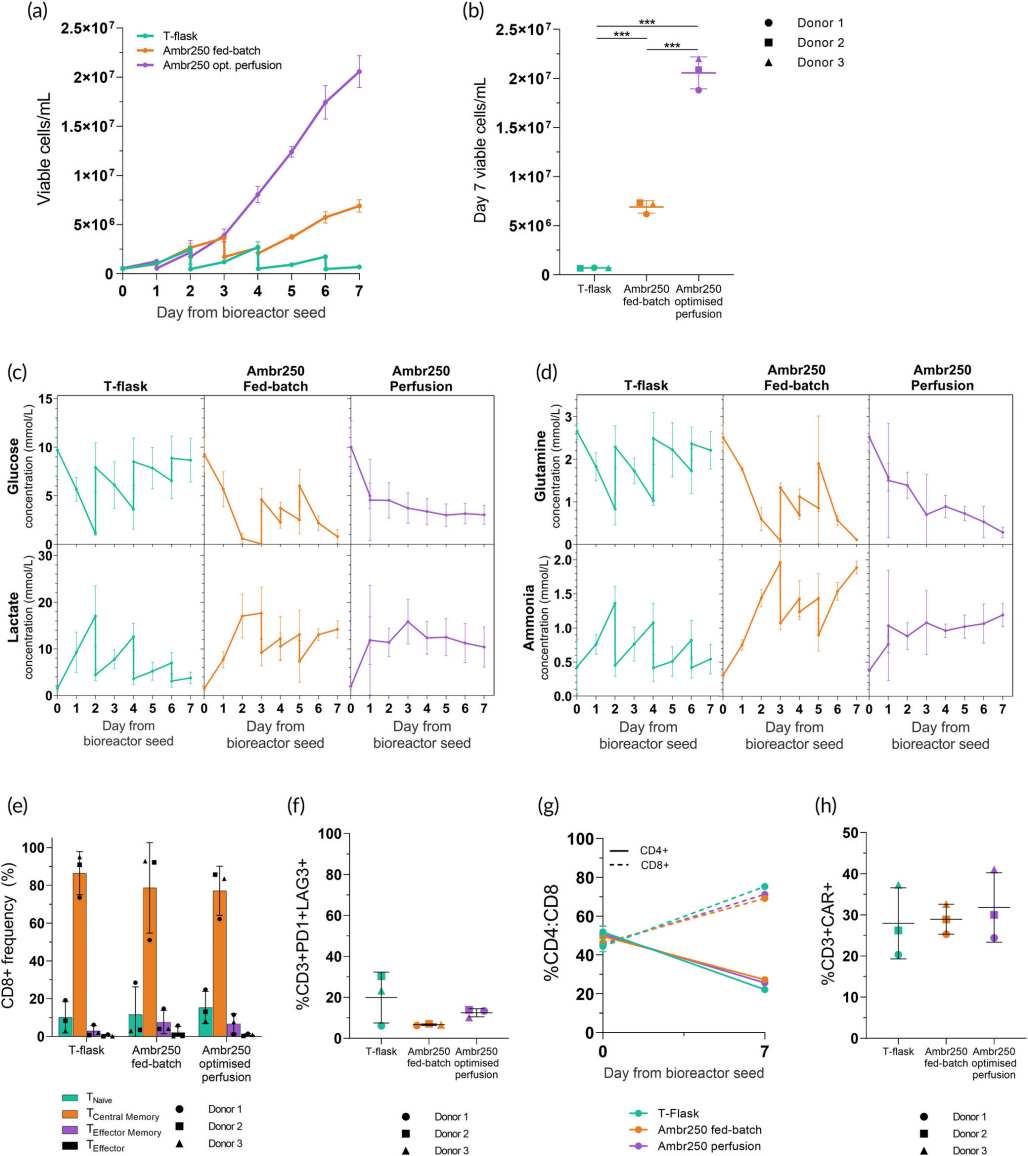
Optimizing perfusion significantly improved cell fold in the Ambr® 250 High Throughput. (a) Viable final cell density by day and (b) by day 7 post‐inoculation. (c) Daily glucose and lactate, and (d) daily glutamine and ammonia concentrations (mmol/L). Media additions were performed on days 3, 4, 5 in the fed‐batch bioreactor, perfusion was started 48 h post‐inoculation at 1 volume vessels per day (VVD) and cell passaging was completed on days 2, 4, 6 in flasks. (e) CD8+ T cell differentiation marker expression at the end of the 7‐day cultures was broken into four populations: %CD8 + CD45RO‐CCR7+ (naïve); %CD8 + CD45RO + CCR7+ (central memory (CM)); %CD8 + CD45RO + CCR7− (effector memory (EM)); and %CD8 + CD45RO‐CCR7‐ (effector). (f) PD1+ and LAG3+ exhaustion marker expression on CD3+ T cells by day 7. (g) Changes in CD4:CD8 ratio from day 0 to 7. (h) CAR expression percentage in harvested CD3+ T cells. Data shown as the mean of *n* = 3 replicates. Error bars represent standard deviation; *p < 0.05, **p < 0.01, ***p < 0.001.

The tested optimized perfusion process resulted in significantly higher CAR‐T cell densities for all three donors compared to both the tested fed‐batch bioreactor and T‐flask processes (Figure 4a). By the final day of culture, the optimized perfusion process yielded 20.6 × 10^6^ ± 1.6 × 10^6^ cells/mL compared with the approximately 7.0 × 10^6^ ± 0.7 × 10^6^ cells/mL in the fed‐batch culture. This represents a nearly 3‐fold and 30‐fold increase in cell density over the fed‐batch bioreactor process and T‐flask process, respectively (Figure 4a). The simultaneous removal of spent medium and replenishment with fresh medium in the optimized perfusion bioreactor processes enabled significantly (*p* < 0.001) higher cell densities to be reached, and this finding was replicated with all three donors (Figure 4b).

Cell growth in each process closely aligned with the observed metabolic trends. In the flask process, metabolite concentrations fluctuated significantly with each passage (every 2 days), and metabolic consumption and production gradually declined alongside cell growth over time (Figure 4c, d). In the fed‐batch process, glucose and glutamine were depleted by day 3 post‐bioreactor seeding and again by day 7, while lactate and ammonia levels steadily accumulated throughout the process. However, initiating perfusion at 1VVD as early as 48 h in the optimized perfusion process prevented glucose and glutamine depletion and stabilized lactate accumulation at approximately 10 mM (Figure 4c, d). These combined effects likely contributed to the observed increase in cell proliferation under perfusion. Interestingly, the consumption of glutamine and associated production of ammonia continued to increase, despite the perfusion of fresh medium at 1 VVD. In the fed‐batch process, delaying the first batch feed of medium until 96 h allowed glucose levels to deplete completely, which negatively impacted cell growth. But even when perfusion was initiated as late as 96 h—resulting in metabolic depletion profiles similar to those in fed‐batch—cell proliferation still almost always outperformed the fed‐batch process by day 7, regardless of the tested perfusion rate (Supplemental Figure 3a, b). This emphasized the advantage of continuous perfusion over intermittent feeding even when initiated at the same time. Additionally, fed‐batch processes are generally constrained by the bioreactor's fixed working volume, limiting the number of batch feeds, whereas perfusion allows flexibility with varying perfusion rates.

Despite achieving significantly higher cell densities, the perfusion bioreactor process yielded cells expressing comparable differentiation and exhaustion marker expression as the fed‐batch and T‐flask processes. Over 90% of harvested CD8+ T cells expressed either naïve (CD45RO‐CCR7+) or central memory (CD45RO + CCR7+) markers (Figure 4e). Less than 5% of harvested CD8+ cells were found to express effector markers (CD45RO‐CCR7‐), despite the significantly higher cell densities achieved in perfusion. Similarly, the flask, fed‐batch, and perfusion bioreactor processes yielded cells expressing exhaustion markers (PD1 + LAG3+) at levels of 19.9%, 6.6%, and 12.5% respectively, differences that were not found to be significant (Figure 4f). Culturing CAR‐T cells to high cell densities in perfusion was therefore not found to be associated with any significant increase in exhaustion marker expression. Exhaustion marker expression was again found to be slightly higher for in CD4+ population compared to CD8+, however, this difference was not found to be significant in either of the three processes. Additionally, cells cultured in fed‐batch and perfusion bioreactors expressed lower and more consistent levels of exhaustion marker expression compared to static T‐flasks, with lower standard deviations of ±0.4%, ±2.0%, and ± 12.4%, respectively. As previously observed, CD4:CD8 ratios consistently shifted from 1:1 to 1:2 over the 7‐day expansion in all three tested processes (Figure 4g) and there were overall no significant differences in CAR expression in either T‐flask or either Ambr® 250 processes (Figure 4h).

### 2.5 Bioreactor CAR‐T cells retain in vitro cytotoxicity capability

**FIGURE 5:**
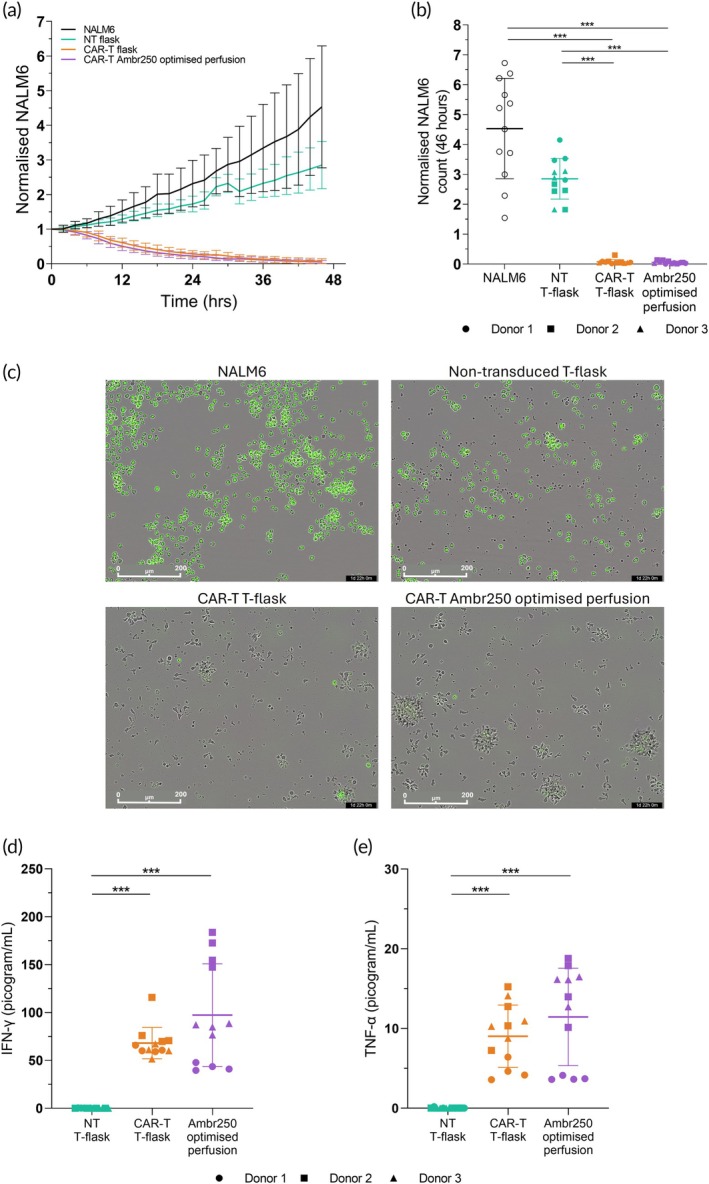
CAR‐T cells remain cytotoxic following perfusion bioreactor expansion. Following expansion in flasks and bioreactor, CAR‐T cells were co‐cultured 1:1 with target Nuclight Green+ NALM6 cells for 2 days. (a) Relative number of NALM6 cells over time and (b) final relative number of NALM6 cells after 2 days, (c) Representative 20X Incucyte® images of CAR‐T cells and controls after 2 days, (d) IFN‐γ and (e) TNF‐α concentration in the medium after 2 days. Data shown as the mean of *n* = 4 replicates. Error bars represent standard deviation; ***p < 0.001. NT, non‐transduced.

An in vitro killing assay was used to assess the CAR‐T cell ability to kill target cells following expansion in the optimized perfusion bioreactor process versus flask cultures. Post‐expansion, cells were co‐cultured with target CD19+ NALM6 cells for 2 days. CAR‐T cells expanded to high cell densities in the perfusion bioreactor retained comparable cytotoxicity as T‐flask controls (Figure 5a). CAR‐T cells from both flask and optimized perfusion bioreactor processes a normalized NALM6 count below 1 for the entirety of the co‐culture, indicating that both CAR‐T cell conditions were able to effectively kill the target cells. In comparison, the NALM6‐only control and co‐culture with non‐transduced T cells showed a continued increase in the NALM6 cells over time (Figure 5a). The co‐cultures with CAR‐T cells that were previously expanded in the flask and optimized perfusion bioreactor cultures all had essentially no NALM6 cells remaining at the end of the 2‐day culture (Figure 5b). This was not specific to the optimized perfusion conditions, as this effective killing was observed for all the perfusion conditions tested in the DOE (Supplemental Figure 5a). Representative microscopy images from the end of each co‐culture illustrate the killing of the nuclear green‐labeled, CD19+ NALM6 cells by CAR‐T cells, which were observed as expanded and elongated in shape, indicating activation in response to the target cells (Figure 5c).

Cytokine analysis of medium samples taken at the end of the 2‐day co‐culture also showed CAR‐T cells from the optimized perfusion process produced comparable levels of IFN‐Ɣ and TNF‐⍺ as the flask process. Perfusion bioreactor and flask CAR‐T cells released significantly more of both cytokines than the non‐transduced control (*p* < 0.001) (Figure 5d, e). Similarly, all CAR‐T cells yielded by different perfusion experiments in the DOE also demonstrated release of IFN‐Ɣ and TNF‐⍺ (Supplemental Figure 5b).

## 3 DISCUSSION

Optimizing perfusion in the Ambr® 250 High Throughput Perfusion bioreactor led to a three‐fold increase in final yields of cytotoxic CAR‐T cells, reaching 22 × 10^6^ cells/mL with no adverse effects on phenotypic characteristics. When comparing to literature, these results align with respect to the positive impact of perfusion on T cell growth kinetics. In a rocking motion bioreactor, Smith et al., demonstrated T‐cell cultures in perfusion reaching approximately 20 × 10^6^ cells/mL^9^, and an additional study by Gatla et al., in STRs also achieved approximately 30 × 10^6^ cells/mL^10^. However, the final cell yields in these studies were achieved over longer expansion times or by starting with a higher number of cells than as described in this work. This emphasizes the importance of optimizing individual perfusion parameters to maximize CAR‐T cell yield within shorter expansion times.

A key challenge in autologous CAR‐T manufacturing remains the patient‐to‐patient variability of the manufacturing starting material. Results in this study have highlighted that optimal feeding regimes differed slightly based on donor. These differences could potentially be amplified when using patient material for autologous products because patient material often has increased variability compared to healthy donor material.^14^ Increased knowledge about the CPPs for feeding during CAR‐T expansion could therefore be utilized to develop an adaptive manufacturing control strategy that could respond to these differences. This concept of adaptive manufacturing is aligned with the concept of implementing QbD approaches to biopharmaceutical manufacturing. Regulators are also promoting this approach, as seen by the FDA's “Pharmaceutical Current Good Manufacturing Practices of the 21st Century—A Risk Based Approach” initiative.^15^

An important aspect for the application of these QbD principles is to utilize Process Analytical Technologies (PAT).^16^ PATs contribute to the design, analysis, and control of manufacturing processes through the timely measurement of critical quality attributes (CQAs) and process parameters to enable the production of a more robust final product.^15^ This concept could be particularly useful for cell therapy manufacturing which typically has variable cell starting material for the manufacturing process. This work found metabolites levels in the medium to be linked to cell growth. Therefore glucose, glutamine, and lactate levels could potentially be used to initiate, monitor, and adapt cell feeds over time. Integration of PATs such as Raman spectroscopy has previously been demonstrated in the Ambr® 250 for online monitoring of glucose and lactate concentrations in a CHO antibody production process.^17^ For perfusion CAR‐T cell cultures, similar monitoring of metabolites in real‐time could allow the feeding process to be adapted to the process needs on an individual donor basis.

The results presented here also underscore the significance of small‐scale STR models, such as the Sartorius Ambr® 250 High Throughput Perfusion bioreactor for the identification and optimization of CPPs. The impact and utility of such scale‐down models have been demonstrated for the development of other biological products including monoclonal antibody production,^18^ human induced pluripotent stem cells,^19^ adeno‐associated virus^20^ and lentiviral vectors.^21^ This technology offers high‐throughput capabilities in small culture volumes while maintaining robust process monitoring and control. Furthermore, it provides a crucial platform for assessing donor‐to‐donor variability in an allogeneic context as part of small‐scale donor screening studies. Ultimately, small‐scale, high throughput models minimize medium requirements during development, reduce time, cost, and risk, and facilitate the incorporation of QbD principles early on in process development.

## 4 MATERIALS AND METHODS

### 4.1 Primary CAR‐T cell production

Primary T cells used in these studies were isolated from healthy, human donors from leukopak (BioIVT, West Sussex, UK) samples. CD3+ T cells were isolated using the human Pan T isolation kit (Miltenyi Biotec Ltd., UK) according to the manufacturer protocol.

The primary T cell culture medium was Roswell Park Memorial Institute (RPMI) 1640 (Gibco™, Thermo Fisher Scientific, UK), 10% foetal bovine serum (Gibco™, Thermo Fisher Scientific, UK), 2 mM L‐glutamine (Gibco™, Thermo Fisher Scientific, UK), and 1% Antibiotic‐Antimycotic (100X, Gibco™, Thermo Fisher Scientific, UK). Primary T cell medium was supplemented with Interleukin‐2 (Miltenyi Biotec, Surrey, UK) at a concentration of 30 IU/mL.

1 day after thaw, the T cells were activated at a 1:1 ratio of cells to Magnetic Dynabeads® (Thermo Fisher Scientific Inc., Waltham, MA, USA) according to the manufacturer protocol. T cells were transduced 1 day later with the CAR lentivirus using a multiplicity of infection of 3 in 6‐well suspension cell culture plates (Sarstedt AG & Co. KG, Germany) coated with RetroNectin® (Takara Bio Inc., France). The CAR lentivirus was produced as previously described^22^ and the functional titre was assessed using an infectivity assay.^23^ The plates were spinnoculated by centrifugation at 1000 *g* for 40 min. 1 day after transduction, cells were washed, plated, and expanded for 3 days until the expansion phase was initiated.

At the start of the expansion phase, cells were plated in fresh medium at a concentration of 0.5 × 10^6^ cells/mL. Daily 1 mL samples were taken for cell counts and metabolite analysis.

### 4.2 STR culture

The expansion of CAR‐T cells both fed‐batch and perfusion was performed in the Ambr® 250 High Throughput STR system (Sartorius, UK). An unbaffled, single‐impeller Ambr® 250 High Throughput vessel^24^ and a custom‐made unbaffled Ambr® 250 High Throughput Perfusion ATF 0.2 μm vessel (Sartorius, UK) were used for fed‐batch and perfusion cultures respectively. Fed‐batch cultures were performed by inoculating 0.5 × 10^6^ cells in 100 mL, followed by a 100% medium top‐up on Day 3 from 100 to 300 mL and further increased to 250 mL on day 4. On day 5, a partial medium exchange was performed by manually removing 100 mL of cultures, resuspending the cells in equal volumes of fresh medium, and returning these to the bioreactor. Associated bioreactor parameters employed in this investigation have been described in a previous study^25^ and included controlling pH and DO at 7.15 and 50% respectively. For perfusion cultures, 50 million cells were first inoculated in 100 mL of media in the STR, and the total working volume was increased to 210 mL, 24 h before the onset of perfusion. Perfusion was initiated at either 48, 72, or 96 h after inoculation at either 0.25, 0.5, or 1 vessel volumes per day (VVD) depending on the experimental condition. Daily samples of 1 mL were taken for offline cell counting and supernatant metabolite analysis. For flask expansion controls, cells were inoculated in static flasks at 0.5 × 10^6^ cells/mL and fed every other day by diluting the culture back down to the same cell density using fresh growth medium. Harvested cells were cryopreserved in CryoStor® CS10 (STEMCELL Technologies, UK) in liquid nitrogen for later analysis.

### 4.3 T cell analytics

Cell density and viability were measured using the NucleoCounter® NC‐3000™ (ChemoMetec A/S©, Denmark) with Via‐1‐Cassettes™ (ChemoMetec A/S©, Denmark) according to the manufacturer's protocol. Medium samples were analyzed using the CuBiAn HT270 bioanalyzer (Optocell GmbH & Co, KG, Germany) to determine levels of glucose, glutamine, ammonia, and lactate concentrations.

Phenotypic activation, differentiation, and exhaustion characteristics were analyzed via flow cytometry on fresh cell samples on the day of sampling using the BD LSRFortessa™ X‐20 flow cytometer (BD Biosciences, UK). Cells were stained for CD3‐BUV395, CD4‐BUV805, CD8‐APC‐Cy7, CCR7‐BV421, CD45RO‐PE‐Cy7, CD56‐BV605, CD34(CAR)‐AlexaFluor647, CD69‐FITC, PD‐1‐PE, LAG‐3‐ BV711, and Live/Dead‐UV511. A minimum of 50,000 events were recorded for all conditions. Gates were confirmed based on fluorescence minus one (FMO) controls for CCR7, CD45RO, CD56, CD69, PD‐1, and LAG‐3.

### 4.4 CAR‐T cytotoxicity and cytokine release assay

The in vitro killing assay was evaluated using the Incucyte® S3 Live‐cell analysis system (Sartorius, UK) according to the manufacturer protocol. A 1:1 target to effector 2‐day co‐culture was used with Incucyte® Nuclight green‐transduced, CD19‐positive NALM6 target cells. One day post‐thaw, the CAR‐positive T‐cells were isolated using a CD34 MicroBead isolation kit (Miltenyi Biotec Ltd., UK). At the end of the 2‐day co‐culture, media supernatant samples were taken for cytokine analysis for tumor necrosis factor‐alpha (TNF‐⍺) and interferon‐gamma (IFN‐Ɣ), performed using the iQue® Human T cell Activation Cell and Cytokine Profiling Kit (Sartorius, UK) and iQue®3 HTS cytometer.

### 4.5 DOEs and statistical analyses

The DOE half‐factorial design, analysis, and data representation were performed using MODDE® v13 (Sartorius, Germany). Data from *n* = 15 cultures were analyzed using an analysis of variance to identify significant main effects and interactions. All coefficients included within the models had a *p*‐value <0.05 (unless they were included to hierarchy for interaction terms), indicating their inclusion in the model was significant. The overall model accuracy and fit for purpose were assessed using *R* ^2^ and *Q* ^2^ values.

Statistical analyses were completed using GraphPad Prism 10 (GraphPad, La Jolla, USA). The variance of the means was determined using the appropriate statistical method of Brown‐Forsythe, Levene, or Bartlett. A comparison of the means was completed by an ANOVA or Welch's ANOVA and, if applicable, a post‐hoc pairwise comparison using Tuckey–Kramer, Steel Dwass, or Dunn pairwise, was then completed.

Summary diagrams in this report were created with BioRender.com (BioRender, Toronto, Ontario). Line charts and bar graphs were completed using GraphPad Prism 10 (GraphPad, La Jolla, USA).

## 5 CONCLUSIONS

These results illustrate the importance of process intensification and identifying and optimizing CCPs such as feeding strategy to maximize CAR‐T expansion. A DOE was completed to understand the impact of the perfusion start time, perfusion rate, and donor variability on CAR‐T expansion in perfusion‐stirred tank bioreactors. The results of this DOE suggested all factors tested impacted cell growth, with perfusion start time and then perfusion rate impacting growth the most. Interestingly, the growth trends were found to be correlated to the depletion of glucose and glutamine, as well as the accumulation of lactate in the culture. This is likely why the perfusion strategy significantly increased cell growth and highlights the benefits of taking a perfusion‐based approach to continuously remove waste products and ensure a constant supply of nutrients to the cells. Within the DOE design space investigated, the perfusion strategy did not have an adverse impact on cell quality or function despite achieving much higher cell densities. Only donor variability impacted the differentiation of the cells, albeit within expected ranges, and no DOE factor resulted in an adverse impact on exhaustion marker expression or killing ability of the CAR‐T cells.

The findings in this study illustrate how using an automated bioreactor with an optimized perfusion feeding process enables reproducible CAR‐T expansion with high cell yields, while significantly reducing process costs, with scope for scalability at the liter‐scale and beyond. Optimizing perfusion supported high‐cell density cultures of cytotoxic CAR‐T cells without any associated increase in terminal differentiation or cellular exhaustion. These results also demonstrate the advantages of having an effective scale‐down model for the manufacturing platform being used for cell expansion, such as the Ambr 250® High Throughput Perfusion. Using such a platform results in significant cost, time, medium, and reagent savings when developing bioreactor processes, and enables incorporation of QbD via DOE to increase process understanding, reduce risk, and support the clinical journey to commercialization. The findings from this study have implications for both autologous CAR‐T manufacture by reducing manufacturing turnaround time and allogeneic manufacture by maximizing yield, reducing footprint, and increasing production capacity for CAR‐T immunotherapies.

## AUTHOR CONTRIBUTIONS

**Tiffany Hood:** Writing – original draft; conceptualization; methodology; investigation; data curation; formal analysis; visualization. **Pierre Springuel:** Writing – review and editing; visualization; writing – original draft; formal analysis. **Fern Slingsby:** Methodology; formal analysis; writing – review and editing. **Viktor Sandner:** Methodology; software. **Winfried Geis:** Methodology; software. **Timo Schmidberger:** Visualization; software; validation. **Nicola Bevan:** Methodology; writing – review and editing. **Quentin Vicard:** Conceptualization. **Julia Hengst:** Project administration. **Noushin Dianat:** Project administration; conceptualization. **Qasim A. Rafiq:** Supervision; funding acquisition; project administration.

## FUNDING INFORMATION

The authors would like to acknowledge the funding and support of UK Engineering and Physical Sciences Research Council (EPSRC) through the Future Targeted Healthcare Manufacturing Hub hosted at University College London with UK university partners (Grant Reference: EP/P006485/1) and includes financial and in‐kind support from the consortium of industrial users and sector organizations. The work was also supported by a UKRI EPSRC Fellowship grant awarded to Professor Qasim Rafiq (EP/V058266/1), by the UCL ORS and GRS schemes which funded Tiffany Hood's studentship, and the EU project AIDPATH (grant agreement number 101016909) which funds Pierre Springuel's studentship.

## CONFLICT OF INTEREST STATEMENT

There are no conflicts of interest to declare for Hood T., Springuel P., and Rafiq Q. A. It should be noted that authors Slingsby F., Sandner V., Geis W., Schmidberger T., Bevan N., Vicard Q., Hengst J., and Dianat N. were or are Sartorius employees during the experimental studies and preparation of the manuscript.

## Supporting information


**Supplemental Figure 1.** DOE design and fold expansion model. (a) Experimental DOE parameters (*n* = 15), (b) fold expansion regression model terms, (c) observed versus predicted fold expansions, (d) regression coefficients fold expansion responses. VVD = vessel volumes per day.

**Supplemental Figure 2.** (a) CD8+ T cell differentiation marker expression at inoculation and harvest of the Ambr® 250 perfusion DOE cultures. Marker expression was broken into four populations: %CD8 + CD45RO‐CCR7+ (naïve); %CD8 + CD45RO + CCR7+ (central memory (CM)); %CD8 + CD45RO + CCR7‐ (effector memory (EM)); and %CD8 + CD45RO‐CCR7‐ (effector). (b) PD1+ and LAG3+ exhaustion marker expression on CD4+ versus CD8+ T cells by day 7. Data represents *n* = 15 perfusion DOE conditions VVD = vessel volumes per day.

**Supplemental Figure 3.** CAR‐T cell growth and metabolite trends for perfusion versus fed‐batch processes initiated 96 hours post‐bioreactor inoculation. (a) Viable cell density by day and (b) daily glucose, lactate, glutamine and ammonia metabolite concentrations. Data represents *n* = 5 perfusion DOE conditions and *n* = 3 fed‐batch runs in the Ambr® 250 bioreactor. Error bars represent standard deviation; VVD = vessel volumes per day.

**Supplemental Figure 4.** Comparison of PD1+ and LAG3+ exhaustion marker expression on CD4+ versus CD8+ T cells by day 7 in the T‐flask, Ambr® 250 fed‐batch and Ambr® 250 optimized perfusion processes. Lines represents the mean of *n* = 3 donor replicates. Error bars represent standard deviation.

**Supplemental Figure 5** CAR‐T killing activity from all 15 DOE perfusion experiments in the Ambr® 250 High Throughput.
Following expansion in T‐flasks and bioreactor, CAR‐T cells were co‐cultured 1:1 with target Nuclight Green+ NALM6 cells for 2 days. (a) Relative number of NALM6 cells over time. (b) IFN‐γ and TNF‐α concentration in the medium after 2 days. Day represents media feed start time. Data shown as the mean of *n* = 4 replicates. Error bars represent standard deviation; ***p < 0.001. VVD = vessel volumes day per day.

## Data Availability

The data that support the findings of this study are available from the corresponding author upon reasonable request.
