# Supplementary figures and images for: Establishing a scalable perfusion strategy for the manufacture of CAR‐T cells in stirred‐tank bioreactors using a quality‐by‐design approach
Source: Bioeng Transl Med. 2025 Jan 28;10(3):e10753. doi: 10.1002/btm2.10753 (PMC12079453; doi:10.1002/btm2.10753)

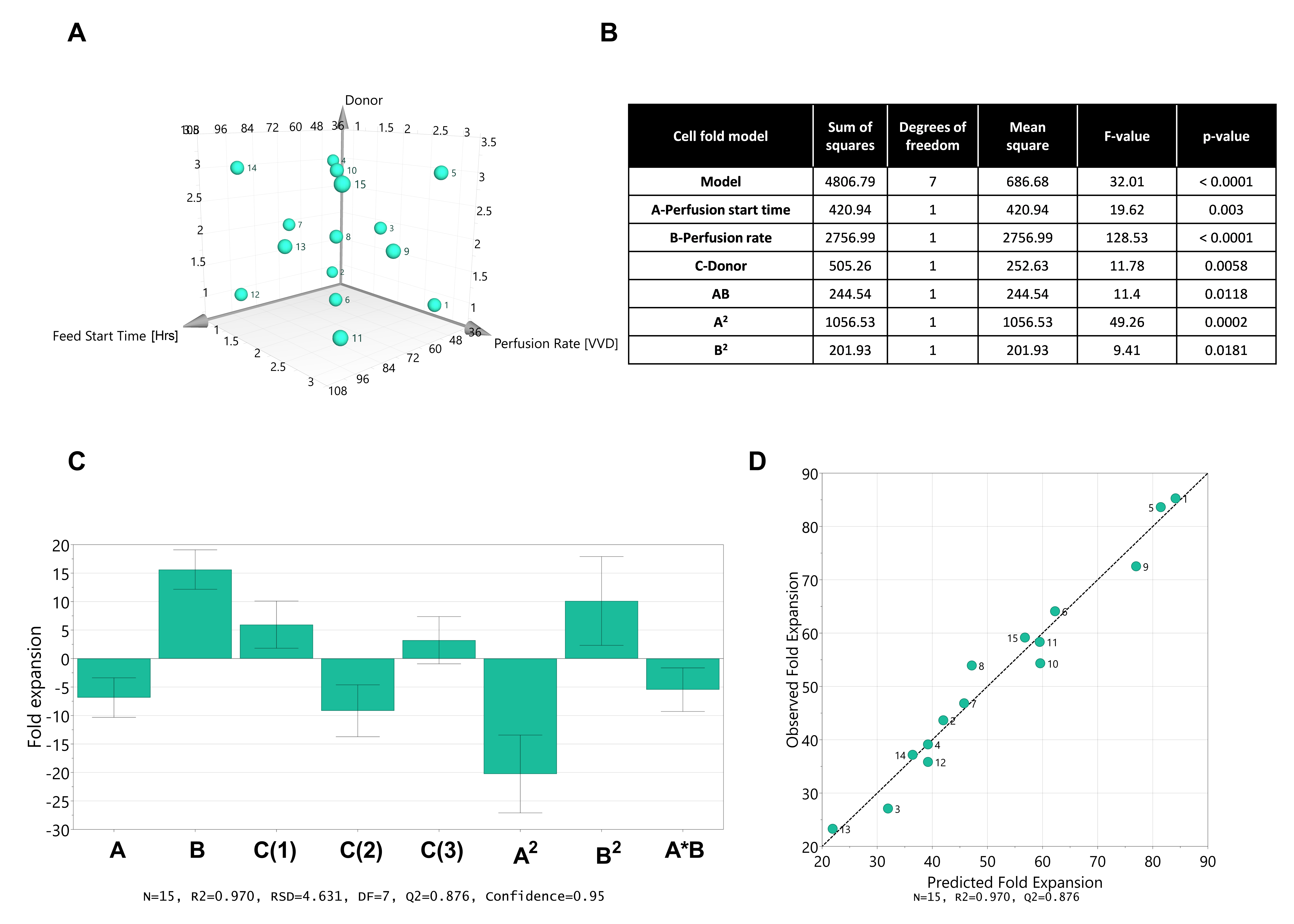

Supplement: Supplementary file 1 — Supplemental Figure 1. DOE design and fold expansion model. (a) Experimental DOE parameters (n = 15), (b) fold expansion regression model terms, (c) observed versus predicted fold expansions, (d) regression coefficients fold expansion responses. VVD = vessel volumes per day. [file BTM2-10-e10753-s001.png]

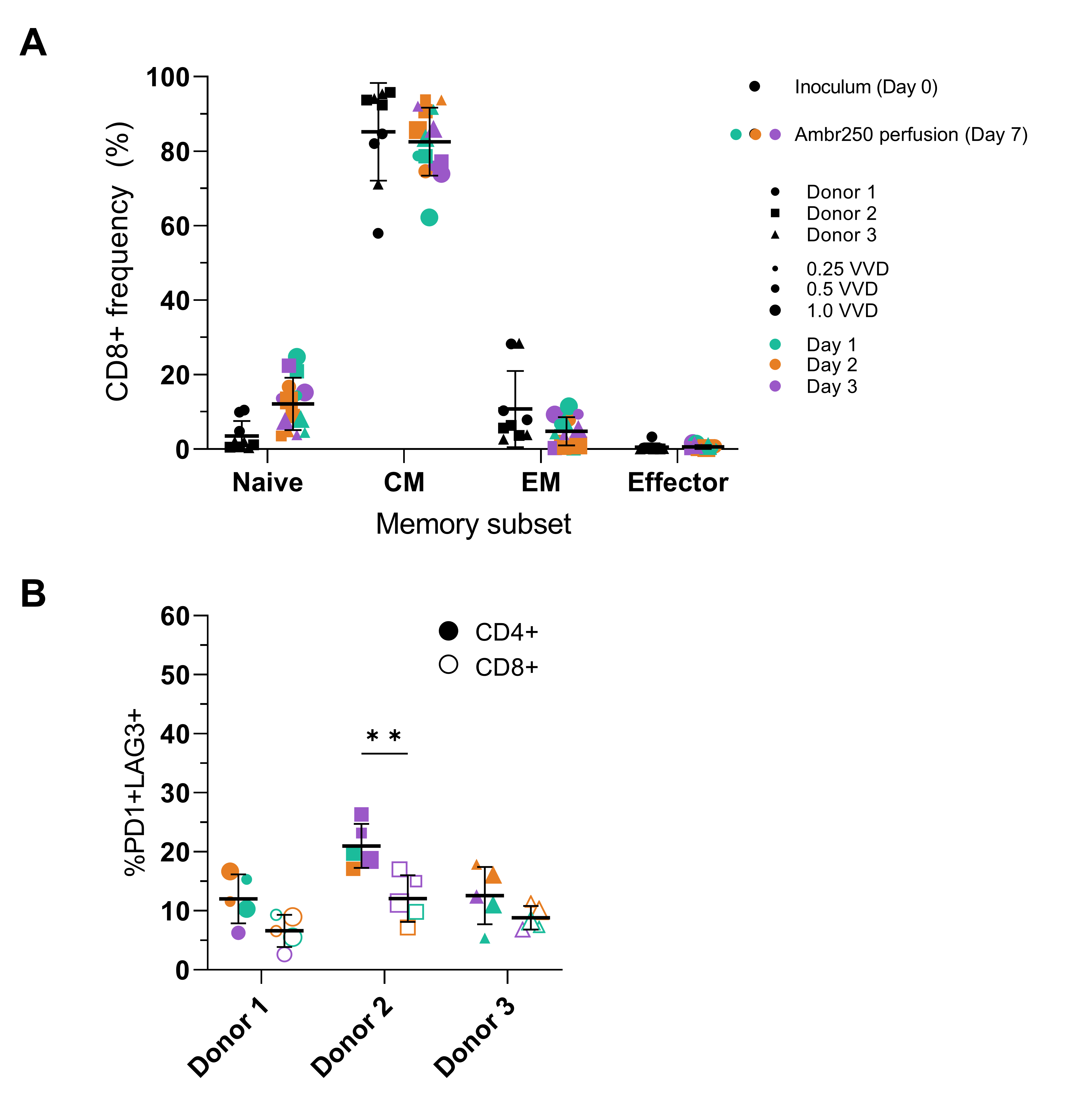

Supplement: Supplementary file 2 — Supplemental Figure 2. (a) CD8+ T cell differentiation marker expression at inoculation and harvest of the Ambr® 250 perfusion DOE cultures. Marker expression was broken into four populations: %CD8 + CD45RO‐CCR7+ (naïve); %CD8 + CD45RO + CCR7+ (central memory (CM)); %CD8 + CD45RO + CCR7‐ (effector memory (EM)); and %CD8 + CD45RO‐CCR7‐ (effector). (b) PD1+ and LAG3+ exhaustion marker expression on CD4+ versus CD8+ T cells by day 7. Data represents n = 15 perfusion DOE conditions VVD = vessel volumes per day. [file BTM2-10-e10753-s002.png]

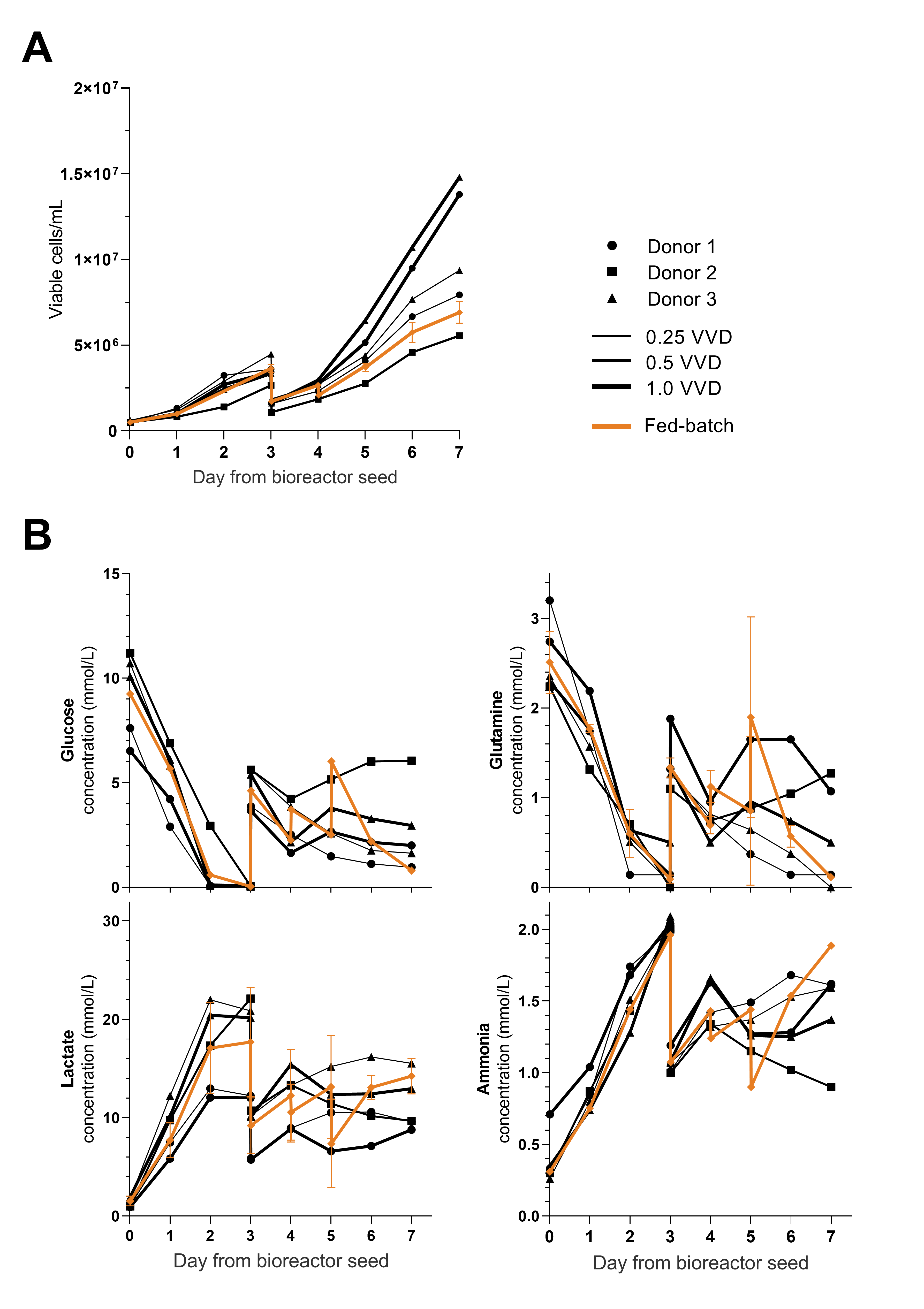

Supplement: Supplementary file 3 — Supplemental Figure 3. CAR‐T cell growth and metabolite trends for perfusion versus fed‐batch processes initiated 96 hours post‐bioreactor inoculation. (a) Viable cell density by day and (b) daily glucose, lactate, glutamine and ammonia metabolite concentrations. Data represents n = 5 perfusion DOE conditions and n = 3 fed‐batch runs in the Ambr® 250 bioreactor. Error bars represent standard deviation; VVD = vessel volumes per day. [file BTM2-10-e10753-s004.png]

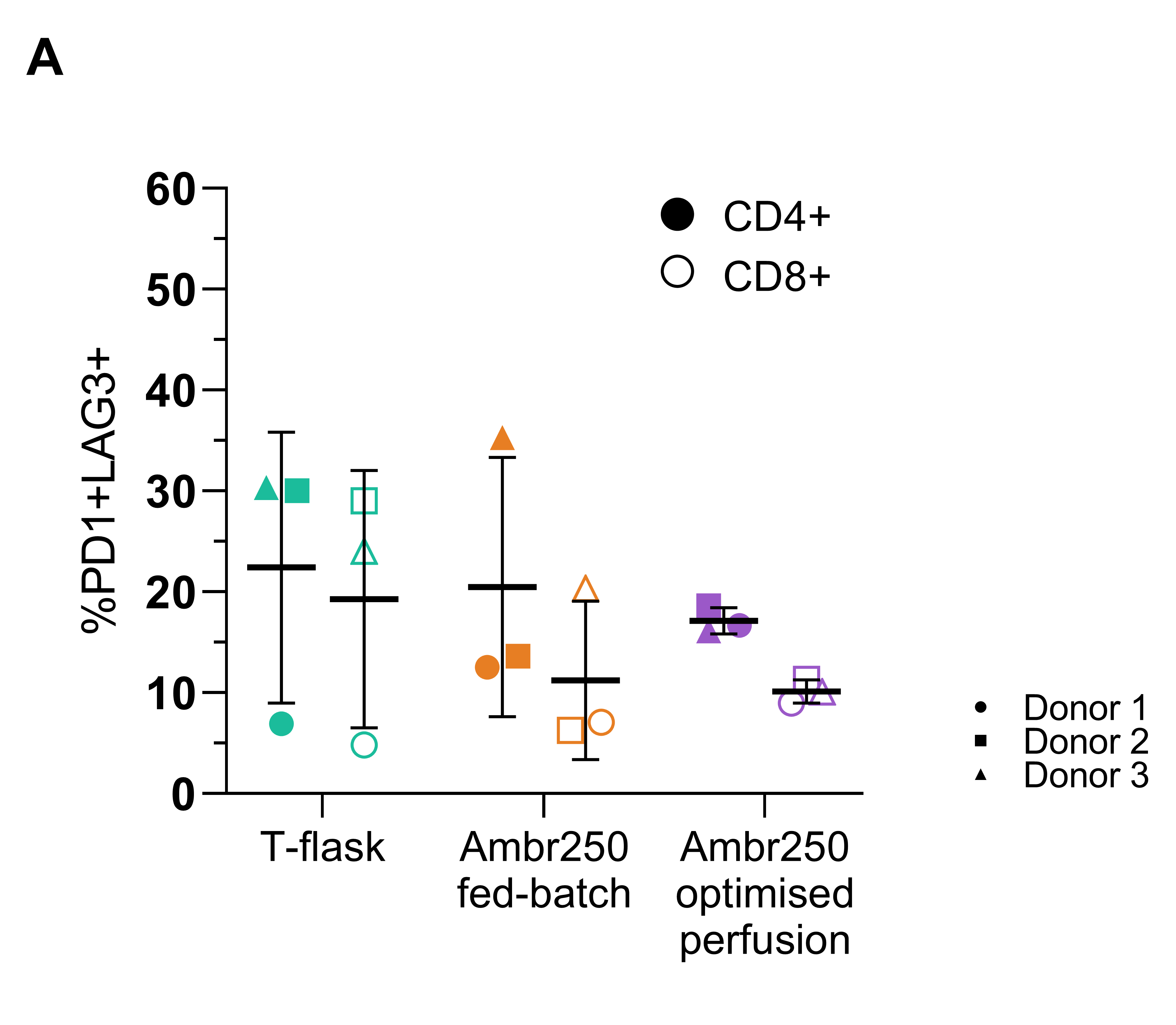

Supplement: Supplementary file 4 — Supplemental Figure 4. Comparison of PD1+ and LAG3+ exhaustion marker expression on CD4+ versus CD8+ T cells by day 7 in the T‐flask, Ambr® 250 fed‐batch and Ambr® 250 optimized perfusion processes. Lines represents the mean of n = 3 donor replicates. Error bars represent standard deviation. [file BTM2-10-e10753-s003.png]

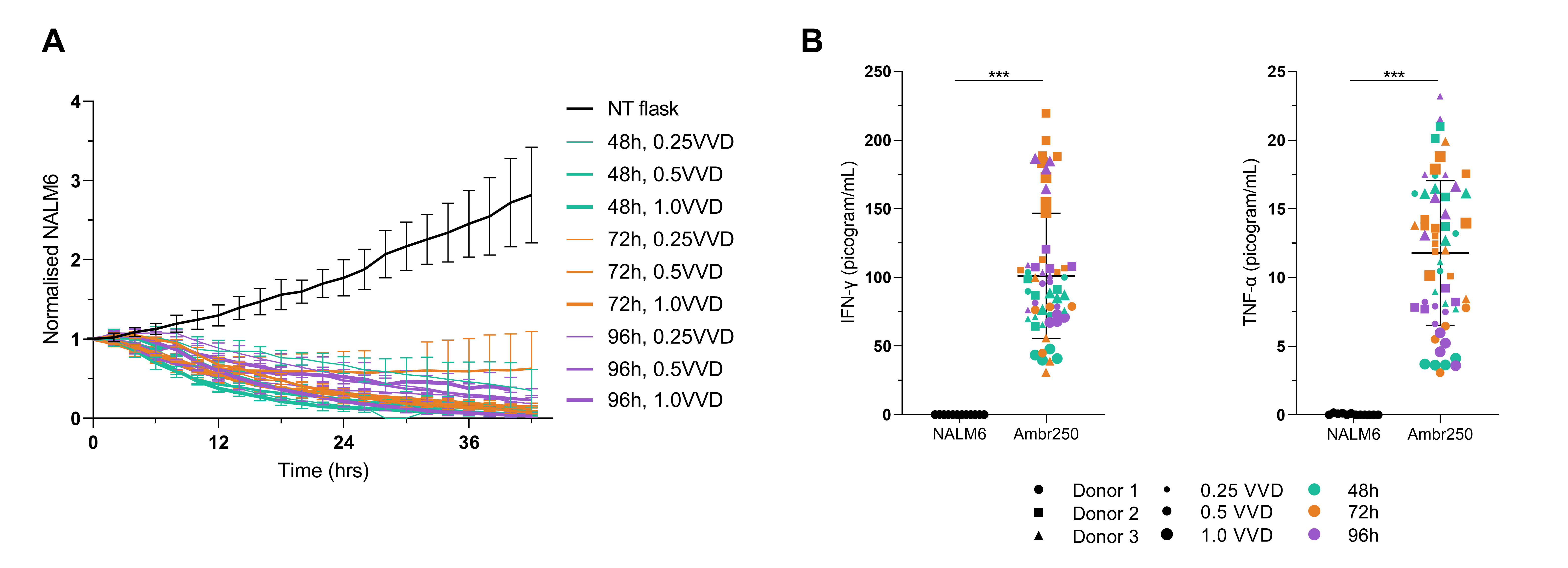

Supplement: Supplementary file 5 — Supplemental Figure 5 CAR‐T killing activity from all 15 DOE perfusion experiments in the Ambr® 250 High Throughput. Following expansion in T‐flasks and bioreactor, CAR‐T cells were co‐cultured 1:1 with target Nuclight Green+ NALM6 cells for 2 days. (a) Relative number of NALM6 cells over time. (b) IFN‐γ and TNF‐α concentration in the medium after 2 days. Day represents media feed start time. Data shown as the mean of n = 4 replicates. Error bars represent standard deviation; ***p < 0.001. VVD = vessel volumes day per day. [file BTM2-10-e10753-s005.png]
